# Severe, eosinophilic asthma in primary care in Canada: a longitudinal study of the clinical burden and economic impact based on linked electronic medical record data

**DOI:** 10.1186/s13223-018-0241-1

**Published:** 2018-04-24

**Authors:** Don Husereau, Jason Goodfield, Richard Leigh, Richard Borrelli, Michel Cloutier, Alain Gendron

**Affiliations:** 1 Department of Epidemiology and Community Medicine, School of Epidemiology and Public Health, University of Ottawa, Room 101, 600 Peter Morand Crescent, Ottawa, ON K1G 5Z3 Canada; 2 IQVIA, Mississauga, ON Canada; 3 Department of Medicine, Cumming School of Medicine, University of Calgary, Calgary, AB Canada; 4 AstraZeneca Canada Inc, Mississauga, ON Canada; 5 Department of Medicine, University of Montreal, Montreal, QC Canada

**Keywords:** Asthma, Severe, Eosinophilia, Interleukin-5

## Abstract

**Background:**

Stratification of patients with severe asthma by blood eosinophil counts predicts responders to anti-interleukin (IL)-5 (mepolizumab and reslizumab) and anti-IL-5 receptor α (benralizumab) therapies. This study characterized patients with severe asthma who could qualify for these biologics in a primary care setting.

**Methods:**

We retrospectively selected patients from July 1, 2010, to June 30, 2014, using a linked electronic medical records (EMR) database (IMS Evidence 360 EMR Canada) for > 950,000 patients in primary care in Ontario, Canada. Patients aged ≥ 12 years with ≥ 2 documented asthma diagnoses were identified as having severe asthma based on prescriptions for high-dosage inhaled corticosteroids (ICS) plus either a leukotriene receptor antagonist, long-acting β_2_-agonist (LABA), or theophylline filled on the same day. Patients’ asthma was considered severe also if they received a prescription for ICS with oral corticosteroids (OCS) or an additional prescription for omalizumab. Patient characteristics, asthma-related medications, and blood eosinophil counts were captured using observed care patterns for the year prior to ICS/LABA and/or OCS prescription. Health care resource use (HCRU) and costs were captured throughout the 1-year follow-up period.

**Results:**

We identified 212 patients who met the criteria for severe asthma. These patients required an average of 6.5 physician visits during the 1-year follow-up period (95% confidence interval 5.7–7.3), and 20 (9%) were referred to respiratory specialists. Overall, 56 patients (26%) with severe asthma had complete blood counts, of whom 23 (41%) had blood eosinophil counts ≥ 300 cells/μL and might be considered for anti-eosinophil therapies. Patients with severe asthma and blood eosinophil counts ≥ 300 cells/μL had more respiratory specialist referrals (17% vs. 12%) than patients with blood eosinophils < 300 cells/μL.

**Conclusions:**

Our data suggest that during 2010–2014, Ontario primary care patients with severe asthma and high blood eosinophil counts had greater HRCU than those with lower counts. Approximately 41% of patients with severe asthma could qualify for anti-eosinophil drugs based on blood eosinophil counts. However, the eosinophilic status of most patients was unknown. It is appropriate to increase awareness of the use of blood eosinophil counts to identify patients who could be considered for anti-eosinophil therapies.

## Background

In Canada, the prevalence of asthma is approximately 8% for patients 12 years and older [1]. Both Canadian and international clinical practice guidelines have defined a spectrum of asthma severity based on the ability to control symptoms with appropriate medications [2–4]. Approximately 5–10% of patients with the disease have severe asthma, which is defined as the need for high-dosage inhaled corticosteroids (ICS) plus a second controller (e.g., long-acting β_2_-agonists [LABA]), and/or oral corticosteroids to control symptoms [5]. Up to 20% of patients with severe asthma have uncontrolled symptoms, which place them at increased risk for diminished health-related quality of life, exacerbations, hospitalizations, and occasional mortality. Moreover, uncontrolled asthma symptoms are associated with significant health care costs [6]. New therapeutics to improve symptom control for patients with severe asthma are necessary.

Increased understanding of the pathophysiology of asthma, as well as emerging biological therapies, has highlighted the need to consider factors beyond symptom control to manage individual patients optimally. The introduction of omalizumab, a humanized anti-immunoglobulin E antibody, highlighted the need to identify patients with an allergic component to their asthma [7]. Furthermore, stratification of patients with severe asthma based on blood eosinophil counts may predict clinical responsiveness to anti-eosinophil therapies, such as mepolizumab and reslizumab (anti-interleukin [IL]-5 antibodies) and benralizumab (anti-IL-5 receptor α antibody) [8–10]. IL-5 is necessary for production, maturation, and survival of eosinophils [11]. Mepolizumab and reslizumab indirectly decrease blood eosinophil counts by binding to and neutralizing circulating IL-5 [12, 13]. Benralizumab binds to the IL-5 receptor on eosinophils and elicits near-complete depletion of eosinophils via enhanced antibody-dependent cell-mediated cytotoxicity [14–16].

The purpose of this study was to characterize and estimate the size of the population of individuals with severe asthma who may require treatment with an anti-eosinophil drug, such as benralizumab, in a primary care setting, along with the clinical and economic burden of their disease. This study was an exploratory analysis using routinely collected data from primary care clinics in Ontario, Canada.

## Methods

### Study aim and design

The primary aim of this study was to determine the prevalence and clinical characteristics of patients with severe asthma who may be responsive to anti-eosinophil therapies in a primary care setting in Canada. It was a retrospective, longitudinal, observational study of patients with severe asthma in primary care in Canada. This study is reported using the REporting of studies Conducted using Observational Routinely collected Data (RECORD) extension to the STrengthening the Reporting of OBservational studies in Epidemiology (STROBE) statement [17, 18].

#### Patient selection

**Fig. 1:**
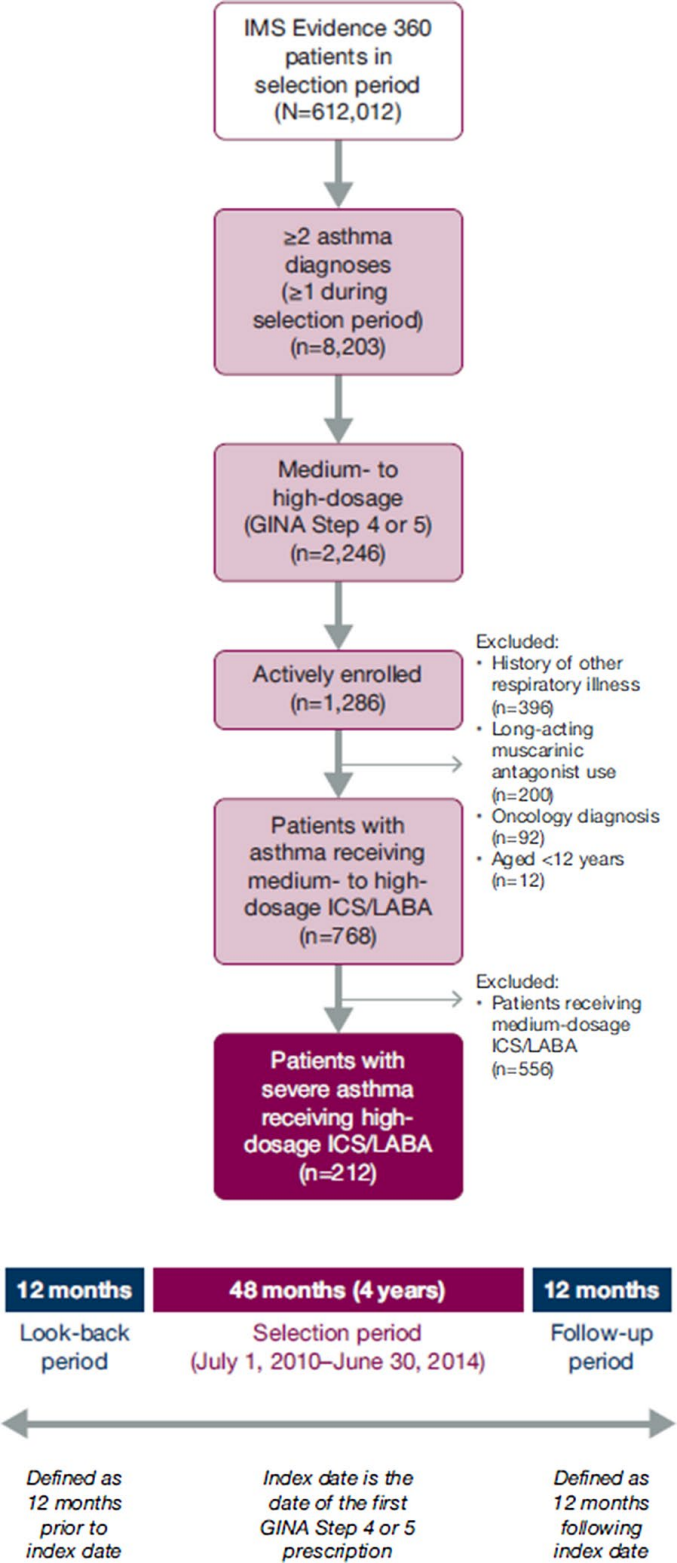
Study design and patient selection. *GINA* Global Initiative for Asthma, *ICS* inhaled corticosteroids, *LABA* long-acting β_2_-agonists

Patients aged ≥ 12 years with ≥ 2 documented asthma diagnoses were retrospectively selected over a 4-year period from July 1, 2010, to June 30, 2014, from a linked electronic medical records (EMR) database (IMS Evidence 360 EMR Canada) containing deidentified longitudinal records for > 950,000 patients in primary care in Ontario, Canada [19]. The database captures approximately 7% of the total population in Ontario, which was 13,680,400 in 2014 [20]. Data from the IMS E360 EMR database are derived from a typical patient visit, including variables such as: year of birth, sex, diagnosis, prescription (including drug identification numbers [1], product, strength, dosage, and refills), lab test results (including blood eosinophil counts), number of visits, specialist referrals, Ontario Health Insurance Plan (OHIP) billing fees, number of times signed off work, and vital signs. Data are loaded from the EMRs on a quarterly basis following deidentification through PARAT software provided by Privacy Analytics Inc. The database has been previously used in the study of chronic diseases in the primary care setting [21, 22]. To account for the progressive nature of asthma, the index date was defined as the date of the first prescription for which severe asthma was observed during the selection period. The follow-up period was defined as the 1-year period commencing on the index date, and the look-back period was defined as the 1-year period preceding the index date (Fig. 1).

Patients included in the study had to satisfy several selection criteria: ≥ 2 asthma diagnoses identified by OHIP diagnosis code 493 (based on International Classification of Diseases, Ninth Revision [ICD-9] diagnosis codes) with at least one diagnosis in the selection period [23, 24]; age ≥ 12 years at index date; active enrollment during both the look-back and the follow-up periods; and severe asthma based on observed medication patterns. Medication patterns classified as indicative of severe asthma were a prescription for high-dosage (as defined by the Global Initiative for Asthma guidelines [3]) ICS along with either a leukotriene receptor antagonist, LABA, or theophylline prescription (from separate prescriptions or a single prescription) filled on the same day. Patients who received a prescription for an oral corticosteroid along with an ICS, or who received an additional prescription for omalizumab were also categorized as having severe asthma. The presence of one of the following criteria in individual patients’ medical histories excluded patients from participating in the study: ≥ 1 long-acting muscarinic antagonist (LAMA) prescription in the 1-year look-back period; or ≥ 1 diagnosis for chronic obstructive pulmonary disease, bronchiectasis, cystic fibrosis, or cancer.

### Variables

Patient demographics, smoking history, comorbidities, asthma-related medications, and blood eosinophil counts were obtained for the year prior to index (look-back period). Prescription of an oral corticosteroid, or prescriptions for short-acting β_2_-agonists (SABA) were captured in the follow-up period. Number of physician visits, laboratory tests, sick notes, and specialist referrals were captured, along with the value of billings by physicians to the OHIP Schedule of Benefits for primary and secondary care. Costs for billing fees are expressed in 2015 Canadian dollars (Can $) based on the Canadian Consumer Price Index for Health Care Services. Data were stratified based on blood eosinophil counts: < 300 and ≥ 300 cells/μL. Emergency department visits and hospital admissions were not included because the data capture in the primary care medical records was not sufficient to create robust estimates.

The list of comorbidities included acute bronchitis (OHIP diagnosis code: 466), rhinitis (OHIP diagnosis code 477), sinusitis (OHIP diagnosis code: 461), anxiety disorders (OHIP diagnosis code: 300), eczema or rash (OHIP diagnosis code: 691), depression (OHIP diagnosis code: 311), diabetes (OHIP diagnosis code: 250), and pneumonia (OHIP diagnosis code: 486) [25–27].

### Clinical burden of disease

Clinical burden of disease was examined by incidence of an emergent (not same-day) prescription of an oral corticosteroid or SABA during the follow-up period. Adherence was measured using the percentage of days covered (PDC) on ICS [28], for all patients received ICS. The denominator for the PDC was the number of days between the first fill of the medication during the follow-up period and the end of the follow-up period. The numerator for the PDC was the number of days covered by the ICS prescription fills during the period covered by the denominator. Patients with a PDC > 80% during the follow-up period were defined as being adherent.

### Statistical methods

Descriptive statistics are presented for patient demographics and clinical characteristics. Categorical variables are expressed in terms of frequency counts and proportions. Continuous variables are expressed in terms of means, medians, and standard deviations. We did not perform formal statistical tests for between-group comparisons.

### Data access and cleaning methods

The investigators had complete access to the IMS E360 EMR database. Data cleaning and correction were performed for the calculations of both body mass index and adherence. As in most databases, weight and height information fluctuations for a given patient were most likely caused by differences in reported units. We performed a correction for a few patients (< 10) to account for high volatility either by inputting the average values for a patient or by converting a value using the appropriate units.

### Linkage

All data were sourced from IMS Evidence 360 EMR Canada. We performed patient-level linkage was performed across output tables using common deidentified patient numbers and visit identification information.

## Results

### Participants

**Fig. 2:**
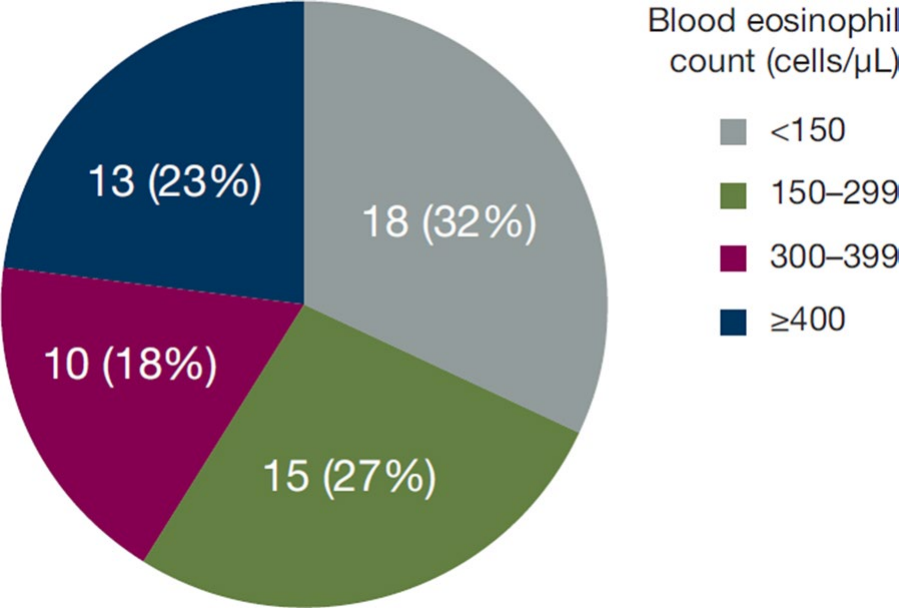
Distribution of blood eosinophil counts. Percentages were calculated based on the 56 patients (26%) with blood eosinophil counts

From the 612,012 patients in the IMS Evidence 360 database during the selection period, 212 patients were identified as having severe asthma and meeting the eligibility criteria. The three most common reasons for patient exclusion were the presence of respiratory comorbidities (specifically chronic obstructive pulmonary disease and bronchiectasis; n = 396 total), use of an LAMA (n = 200), and receipt of medium-dosage ICS/LABA (n = 556; Fig. 1). Fifty-six study-eligible patients (26%) had data for blood eosinophil counts: counts were < 300 cells/μL for 33 of 56 patients (59%) and ≥ 300 cells/μL for 23 of 56 patients (41%) (Table 1, Fig. 2).

**Table 1:** Demographics and baseline clinical characteristics of patients with severe asthma during the 1-year look-back period

	All patients with severe asthma	Patients with severe asthma stratified by eosinophil counts	
		< 300 cells/µL	≥ 300 cells/µL
	N = 212	n = 33	n = 23
Mean age, years (SD)	43 (16)	48 (15)	51 (12)
Aged 12–17 years, n (%)	4 (2)	0 (0)	0 (0)
Aged 18–34 years, n (%)	69 (33)	9 (27)	1 (4)
Aged 35–64 years, n (%)	119 (56)	21 (64)	19 (83)
Aged ≥ 65 years, n (%)	20 (9)	3 (9)	3 (13)
Female, n (%)	124 (58)	25 (76)	14 (61)
Smoking history, n (%)			
Current	42 (20)	4 (12)	0 (0)
Previous	23 (11)	7 (21)	3 (13)
Nonsmoker	138 (65)	19 (58)	19 (83)
Unknown	9 (4)	3 (9)	1 (4)
BMI, mean (SD)	29.2 (6.8)	29.7 (8.0)	29.6 (5.7)
< 18, n (%)	3 (1)	1 (3)	0 (0)
18–24, n (%)	32 (15)	10 (30)	5 (22)
25–29, n (%)	39 (18)	7 (21)	6 (26)
≥ 30, n (%)	45 (21)	11 (33)	9 (39)
Unknown, n (%)	93 (44)	4 (12)	3 (13)
Respiratory specialist referral, n (%)	43 (20)	3 (9)	2 (9)
Mean blood eosinophil count, cells/μL (SD)	303 (266)	140 (79)	537 (267)
Comorbidities, n (%)			
Acute bronchitis	35 (17)	6 (18)	6 (26)
Rhinitis	19 (9)	3 (9)	4 (17)
Sinusitis	19 (9)	5 (15)	2 (9)
Anxiety disorders	19 (9)	3 (9)	9 (39)
Eczema or rash	14 (7)	4 (12)	3 (13)
Depression	7 (3)	1 (3)	0 (0)
Diabetes	15 (7)	5 (15)	5 (22)
Pneumonia	5 (2)	2 (6)	0 (0)
Medications, n (%)			
ICS/LABA^a^	70 (33)	13 (39)	8 (35)
ICS^a^	34 (16)	6 (18)	9 (39)
OCS	18 (8)	3 (9)	1 (4)
LTRA	11 (5)	1 (3)	2 (9)
LABA	0 (0)	0 (0)	0 (0)
Injectable CS	0 (0)	0 (0)	0 (0)
Xanthines	0 (0)	0 (0)	0 (0)
Omalizumab	0 (0)	0 (0)	0 (0)
SABA	88 (42)	13 (39)	9 (39)

*BMI* body mass index, *CS* corticosteroid, *ICS* inhaled corticosteroid, *LABA* long-acting β_2_-agonist, *LTRA* leukotriene receptor antagonist, *OCS* oral corticosteroid, *SABA* short-acting β_2_-agonist, *SD* standard deviation

^a^Medication use recorded during the look-back period. All patients had ICS/LABA at index date

### Characteristics of patients with severe asthma and greater blood eosinophil counts

Patients with severe asthma and eosinophil counts ≥ 300 cells/μL vs. < 300 cells/μL were more often male (39% vs. 24%), nonsmokers (83% vs. 58%), and recipients of ICS prescriptions (39% vs. 18%). Patients with eosinophil counts ≥ 300 cells/μL were also more likely to have acute bronchitis, rhinitis, and anxiety disorders than those with eosinophil counts < 300 cells/μL (look-back period; Table 1).

### The clinical and economic burden of severe asthma

During the follow-up period, 54 of 212 patients (25%) were classified as adherent to ICS therapy (follow-up period; Table 2). Of 212 patients with severe asthma receiving high-dosage ICS/LABA, 23 patients (11%) required new prescriptions for oral corticosteroids during the follow-up period, and 138 patients (65%) were prescribed a SABA. In addition, patients with severe asthma using high-dosage ICS/LABA visited physicians 6.5 times (95% confidence interval [CI] 5.7–7.3) on average during the 1-year follow up. Furthermore, 20 of 212 patients (9%) were referred to respiratory specialists. The average value of physician billing was Can $369 (standard deviation: Can $373).

**Table 2:** Clinical and economic burden of severe asthma during the 1-year follow-up period

	All patients with severe asthma	Patients with severe asthma stratified by eosinophil counts	
		< 300 cells/µL	≥ 300 cells/µL
	N = 212	n = 33	n = 23
New prescriptions, n (%)			
OCS	23 (11)	5 (15)	2 (9)
SABA	138 (65)	20 (61)	11 (48)
Physician visits, mean (SD)	7 (6)	10 (8)	10 (7)
Respiratory specialist referral, n (%)	20 (9)	4 (12)	4 (17)
Number of laboratory tests, mean (SD)	18 (36)	34 (51)	46 (51)
Number of sick notes, mean (SD)	0.3 (1.1)	0.5 (1.6)	0.3 (0.7)
Physician billing [Can $], mean (SD)	369 (373)	517 (396)	620 (646)

*Can $* Canadian $, *OCS* oral corticosteroid, *SABA* short-acting β_2_-agonist, *SD* standard deviation

Compared with patients with severe asthma (using high-dosage ICS/LABA) and eosinophil counts < 300 cells/μL, those with eosinophil counts ≥ 300 cells/μL required more respiratory specialist referrals (17% vs. 12%), incurred greater physician billing costs (Can $620 vs. Can $517) and more laboratory tests (46 vs. 34) (follow-up period; Table 2). However, a smaller percentage of those with eosinophil counts ≥ 300 cells/µL than of those with eosinophil counts < 300 cells/µL received new prescriptions for oral corticosteroids or SABAs. The corresponding median cost (interquartile range) for patients with eosinophil counts ≥ 300 cells/µL and < 300 cells/µL was Can $389 (Can $304–719) vs. Can $371 (Can $232–766).

## Discussion

Our study demonstrates that patients with severe asthma receiving high-dosage ICS/LABA regularly visit primary care clinics in Ontario and use health care resources. These patients required more than the average number of physician visits and referrals to respiratory specialists. Of patients with available complete blood count data, we estimated that 41% would qualify for new therapies that target eosinophil-mediated inflammation based on high blood eosinophil counts (≥ 300 cells/µL). Patients with greater blood eosinophil counts also used more physician and laboratory health care resources than those with counts < 300 cells/µL. Complete blood counts (CBC; and by implication, eosinophil counts) were performed only on a quarter of patients with severe asthma in primary care, yet a relatively high percentage of patients with CBC data (41%) had peripheral blood eosinophilia. Thus, few patients with severe asthma and blood eosinophilia are being identified and referred to respiratory specialists.

We are not aware of any other real-world Canadian studies that have characterized disease burden for patients with severe asthma. However, our study is consistent with previous studies that have estimated the clinical, economic, and individual patient-reported burden of disease for Canadians with broader diagnoses of asthma. For example, our estimate of 7 physician visits annually sits midrange between estimates of 4–17 visits in other studies of adult patients with severe asthma [29]. It is similar to an average of 7.5 (95% CI 7.1–7.9) reported visits in a cohort of Quebec-based patients with asthma requiring combination LABA with high-dosage (mean 543-μg fluticasone equivalent) ICS therapy [30]. In contrast, a British Columbia cohort that was identified as being inappropriately managed with high SABA and low ICS use (≥ 9 canisters of SABA per week and ≤ 100 μg/d of ICS) reported 17 (95% CI 115–118) visits [31].

Furthermore, we are not aware of other Canadian studies that have attempted to characterize the burden of asthma for patients with severe, eosinophilic asthma. Although there is still some uncertainty as to the value of using a single test to characterize the severity of eosinophilic inflammation [32–34], patients with blood eosinophil counts ≥ 300 cells/µL benefit from anti–IL-5 and anti–IL-5 receptor α therapy for reducing steroid use and exacerbations and improving control of asthma symptoms [16, 17, 35–37]. The percentage of patients with very high eosinophil counts (≥ 400 cells/µL) in our study was 23%, which is consistent with several other observational studies (16–26%) [38–41] that have also demonstrated a greater rate of symptoms of poor control, exacerbations, and hospitalizations associated with greater eosinophil counts.

One strength of our approach is that it is based on a large sample of patients and uses a single data structure, which avoids errors related to data linkage. We also used validated algorithms for identifying patients with asthma from administrative data [24, 42] and excluding patients with other diagnoses, including patients using LAMA, which until recently have been recommended only for those with chronic obstructive pulmonary disease. Although new guidance suggests these can now be used in patients with severe asthma with frequent exacerbations [3], the guidance appeared after the selection and follow-up periods for our study. The use of stricter criteria, strengthened the precision of the selection algorithm, but the stricter criteria likely excluded some patients with asthma, particularly those with comorbid respiratory conditions or less severe asthma. In addition, in the absence of direct enrollment indicators, patients were required to have completed visits before their look-back period and following the analysis period. Although the study design allocated at least 1 year of data to detect these confirmatory visits before and after the study period, there is potential for exclusion of patients who do not regularly see their physician, or patients with more severe asthma who are treated by specialists or general practitioners outside the database capture. Nevertheless, we think the selection criteria in our study identified patients most likely to have asthma and with factors associated with poor asthma control.

We made additional effort to minimize systematic errors or bias from confounding our estimates. For example, we chose to use PDC instead of a medication possession ratio (MPR) to estimate medication adherence. PDC provides a more conservative estimate of adherence and is preferred over MPR when patients frequently switch medications or use several medications concurrently within a drug class [43]. Also, we captured patients with eosinophil counts during the 1-year look-back period only, versus both the look-back and follow-up period, to reduce potential confounding from the ordering of tests that are the result of symptom exacerbations. This method resulted in an approximately 20% smaller group of patients for analysis. We also captured medicines prescribed only during the look-back period rather than during the patient’s full history to standardize comparisons across patients with variable lengths of history in the electronic medical records.

One potential limitation of our study is that it includes only primary care physician visits and does not capture patients who see emergency department physicians, respirologists, internists, or other health care specialists. However, a previous study of asthma burden in Ontario showed that 86% of claims to OHIP for nonpediatric asthma were from general practitioners and family physicians—few individuals received care from specialist physicians [44]. Another limitation is that this patient sample was taken from a network of clinics that serve rostered and walk-in patients. In comparison with the general population, patients attending such walk-in clinics have been demonstrated to be younger and healthier with respect to chronic conditions, and to lack regular visits to a family doctor [45]. It is possible that the clinical and economic burden observed for our sample would be greater for a broader patient population receiving routine care in different settings.

The use of electronic medical records from a primary care clinic also limits measures of adherence and laboratory data. Adherence in this study was captured by observing the initial prescription and does not capture whether the medication was subsequently dispensed and whether the patient then used the medication accordingly. Also, most patients were classified based on a single blood eosinophil measurement during the look-back period. As mentioned above, the value of using a single test to characterize the severity of eosinophilic inflammation is not certain. It is also possible that use of systemic corticosteroids during the look-back period may have influenced eosinophil measurements, particularly for the severe cohort, in which 19% of patients had a history of OCS use.

Our study suggests that up to 41% of patients with severe asthma could qualify for new anti-IL-5 and anti-IL-5 receptor α therapies based on a single blood eosinophil count. Accepting an 8% prevalence of asthma in adults [1], of whom a maximum of 10% are estimated to have severe disease [5], and that 25% of these patients with severe disease are not able to achieve control [46, 47], up to 11,218 (13,680,400 × 0.08 × 0.1 × 0.25 × 0.41) patients in Ontario could be considered for these therapies. The actual numbers will likely be much lower given that many patients are likely nonadherent to their maintenance medications, as confirmed in our study, and can be controlled with first- or second-line medicines.

## Conclusions

Our data suggest that patients with greater blood eosinophil counts use more health care resources compared with those with lower eosinophil counts. Blood eosinophil counts were performed for only a quarter of patients with severe asthma in primary care. However, of these patients, approximately 41% had blood eosinophil counts ≥ 300 cells/µL and might be considered for anti-eosinophilic therapies. It is necessary to increase awareness of the use of blood eosinophil counts to identify patients who could qualify for anti-eosinophil therapies.

## Abbreviations

BMI: body mass index
Can $: Canadian dollars
CBC: complete blood counts
CI: confidence interval
CS: corticosteroid
DIN: drug identification numbers
EMR: electronic medical records
GINA: Global Initiative for Asthma
HCRU: health care resource use
ICD-9: International Classification of Diseases, Ninth Revision
ICS: inhaled corticosteroids
IL-5: interleukin-5
LABA: long-acting β_2_-agonist
LAMA: long-acting muscarinic antagonist
LTRA: leukotriene receptor antagonist
MPR: medication possession ratio
OCS: oral corticosteroids
OHIP: Ontario Health Insurance Plan
PDC: proportion of days covered
RECORD: REporting of studies Conducted using Observational Routinely collected Data
SABA: short-acting β_2_-agonist
SD: standard deviation
STROBE: STrengthening the Reporting of OBservational studies in Epidemiology
